# Influence of precedent drug on the subsequent therapy in the sequence of trifluridine/tipiracil with/out bevacizumab and regorafenib for unresectable or recurrent colorectal cancer

**DOI:** 10.1371/journal.pone.0269115

**Published:** 2022-06-02

**Authors:** Kotoe Oshima, Hidekazu Hirano, Hirokazu Shoji, Satoru Iwasa, Natsuko Okita, Atsuo Takashima, Narikazu Boku

**Affiliations:** 1 Department of Gastrointestinal Medical Oncology, National Cancer Center Hospital, Tokyo, Japan; 2 Department of Medical Oncology and General Medicine, IMS Hospital, Institute of Medical Science, University of Tokyo, Tokyo, Japan; Seoul National University College of Pharmacy, REPUBLIC OF KOREA

## Abstract

**Background:**

Trifluridine/tipiracil (TFTD), with or without bevacizumab (Bev), and regorafenib are salvage chemotherapy options for metastatic colorectal cancer (mCRC). Here, we examined the influence of precedent drug on the efficacy of subsequent drug.

**Method:**

The subjects were patients with mCRC who received salvage chemotherapy with TFTD (with/without Bev) followed by regorafenib (TFTD→Rego group/TFTD+Bev→Rego group), or reverse sequence (Rego→TFTD group) at the National Cancer Center Hospital between November 2013 and December 2020. The overall survival (OS), progression-free survival (PFS), disease control rate (DCR), tumor growth rate (TGR), and tumor growth kinetics (TGK) in the first evaluation were assessed in the three groups.

**Results:**

A total of 69 patients, including 27 in the TFTD→Rego group, 13 in the TFTD+Bev→Rego group, and 29 in the Rego→TFTD group, were identified. There were no significant differences in the OS among the three groups, and in the PFS and DCR between the precedent and subsequent therapies in any of the groups. The median TGR (%/month) and TGK (mm/month) in the precedent→subsequent therapy were 50.9→32.7 (p = 0.044) and 8.76→7.79 in the TFTD→Rego group, 25.4→36.1 and 7.49→9.92 in the TFTD+Bev→Rego group, and 40.8→24.4 (p = 0.027) and 8.02→7.20 in the Rego→TFTD group, respectively.

**Conclusion:**

In crossover use of TFTD with/without Bev and regorafenib, both agents showed similar efficacy in terms of the conventional parameters, but the differences observed in the TGR and TGK might suggest some influence of prior regorafenib treatment on the efficacy of subsequent TFTD therapy, and vice versa.

## Introduction

Colorectal cancer (CRC) is the third most common cancer and the fourth leading cause of cancer-related death worldwide [1]. Systemic chemotherapy using fluoropyrimidine plus either oxaliplatin (FOLFOX) or irinotecan (FOLFIRI) in combination with anti-vascular endothelial growth factor (VEGF) antibodies or anti-epidermal growth factor receptor (EGFR) antibodies in the case of *RAS* wild-type tumors [2] have been recognized as the standard first- and second- line treatments for patients with metastatic CRC (mCRC)

Regorafenib is an oral multi-kinase inhibitor of multiple pathways involved in angiogenesis, oncogenesis and the tumor microenvironment [3]. A randomized phase III (CORRECT) trial revealed a survival benefit of regorafenib compared with placebo [4]. TFTD is an oral nucleotide antitumor agent consisting of trifluridine (thymidine-based nucleic acid analogue) and tipiracil hydrochloride (thymidine phosphorylase inhibitor) [5, 6]. A randomized phase III (RECOURSE) trial also showed a survival benefit of TFTD compared with placebo [7]. Recently, combination of TFTD and bevacizumab (Bev) demonstrated promising results as compared to TFTD monotherapy in a phase II trial [8], showing median progression-free survival (PFS) of 4.6 months in TFTD plus Bev vs. 2.6 months of TFTD alone.

Thus, TFTD (with/without Bev) and regorafenib are treatment options for salvage chemotherapy for mCRC in clinical practice. Several retrospective studies have shown similar overall survival (OS), PFS and response rates to TFTD and regorafenib in mCRC patients regardless the sequence of administration [9–12]. Although it is recommended to use both available drugs as the treatment strategy, optimal sequence of them has not been established, and the influence of the precedent drug on the efficacy of subsequent drug has not been clarified.

The RECIST guides assessment of changes in the tumor sizes of target lesions, and accordingly, the responses are classified into four categories: complete response (CR), partial response (PR), stable disease (SD), and progressive disease (PD) [13]. As the RECIST guideline does not take into consideration the time between two assessment points, it does not evaluate the impact of treatment on the kinetics of tumor growth [14]. Recently, there have been some reports on assessment of the treatment efficacy based on the kinetics of tumor growth; e.g., the tumor growth rate (TGR) representing changes of the tumor volume over time [15] and the tumor growth kinetics (TGK) representing changes of the tumor size over time [16]. Previous reports have shown the usefulness of TGR for quantitative and dynamic evaluation of the tumor response [17].

This study investigated the influence of precedent drug on the efficacy of subsequent drug in both sequences using TFTD (with/without Bev) and regorafenib in patients with mCRC in terms of various efficacy parameters, including the TGR and TGK.

## Materials and methods

### Patients

The subjects of this study were mCRC patients who received both TFTD (with/without Bev) and regorafenib as salvage line treatments regardless their sequence at the National Cancer Center Hospital between November 2013 and December 2020. The patient selection criteria were; 1) unresectable or recurrent colorectal adenocarcinoma with measurable lesions; 2) Eastern Cooperative Oncology Group (ECOG) performance status (PS) 0–2; 3) refractory or intolerant to fluoropyrimidine, oxaliplatin, irinotecan, anti-vascular endothelial growth factor (VEGF) antibody, and anti-epidermal growth factor receptor antibody (if *KRAS* wild-type); 4) no prior use of TFTD or regorafenib; 5) sequential use of TFTD and regorafenib immediately after the precedent agent; 6) no combination with other drugs than Bev; 7) assessment tumor response of TFTD and regorafenib by CT.

### Treatment

Patients received oral TFTD 35 mg/m^2^ twice daily on day1-5 and 8–12 every 28 days with or without intravenous Bev 5 mg/kg biweekly followed by oral regorafenib 160 mg once daily for the first 3 weeks of each 4week cycle or reverse sequence. These treatments were continued until disease progression, unacceptable toxic effects, or decision to discontinue by the patients or the investigators. Dose reduction and treatment delay were performed by the decision of the investigators.

### Collected clinical data

The subjects were categorized into four groups as follows: precedent TFTD monotherapy followed by subsequent regorafenib (TFTD→Rego group), precedent TFTD plus Bev followed by regorafenib (TFTD+Bev→Rego group), precedent regorafenib followed by subsequent TFTD (Rego→TFTD group), and precedent regorafenib followed by subsequent TFTD plus Bev (Rego→TFTD+Bev group).

The responses were evaluated according to the RECIST version 1.1. The overall response rate (ORR) and disease control rate (DCR) were defined as the proportion of patients showing CR or PR, and those showing CR, PR or SD, respectively.

The TGR was defined as follows. Tumor size (*D*) was defined as the sum of the longest diameters of the target lesions as per the RECIST version 1.1. *t* is the interval (in months) between two CT examinations conducted for tumor evaluation, and assuming that the tumor growth follows the exponential law, *V*_*t*_ = *V*_0_*exp*(*TG*∙*t*), where V_0_ is the tumor volume at the baseline, V*t* is the tumor volume at time *t*, and TG is the growth rate. We approximated the tumor volume (V) by *V* = 4*πR*^3^/3, where R is the radius of one virtual sphere having a diameter equal to the sum of diameters of the target lesions. TG was calculated as follows: *TG* = 3*Log*(*D*_*t*_/*D*_0_)/*t*. TGR is expressed as a percent increase in the tumor volume during a period of 1 month (%/month): *TGR* = 100[*exp*(*TG*)−1] [15]. TGR was calculated using the data between CT images obtained just before and after the initiation of each therapy. TGK was defined as the change in the sum of the diameters of the target lesions per unit time, and it was calculated as the slope between nadir and progression in the original report [16]. In this study, same calculation method was applied to calculate TGK by comparing the data between CT images obtained just before and after the initiation of each therapy. TGR and TGK were assessed by the same investigator.

PFS was defined as the time from the initiation of each agent to the first radiologic confirmation of disease progression or death from any cause. Two-drug PFS (T-PFS) was calculated from initiation of the precedent therapy to PFS event in the subsequent therapy, meaning failure of both drugs. OS was defined as the time from initiation of precedent therapy to death from any cause.

### Statistical analysis

The categorical patient characteristics were compared among the groups using *Fisher’s* exact test. PFS and OS was estimated using the Kaplan–Meier method, and compared by the log-rank test. The TGR and TGK values were compared by the Mann-Whitney *U* test, and also compared between the precedent and subsequent therapy in each group using the Wilcoxon signed-rank test. All analyses were two-tailed, with p <0.05 considered as denoting statistical significance. All statistical procedures were performed using EZR (Saitama Medical Center, Jichi Medical University, Saitama, Japan) [18].

The present study was approved by the institutional Ethics Committee of the National Cancer Center Hospital (approval number; 2017–229), and conducted according to the guidelines for biomedical research specified in the Declaration of Helsinki. Informed consent was not obtained from the patients due to the retrospective nature of this study. All data were fully anonymized before we accessed them.

## Results

### Patient characteristics

**Fig 1:**
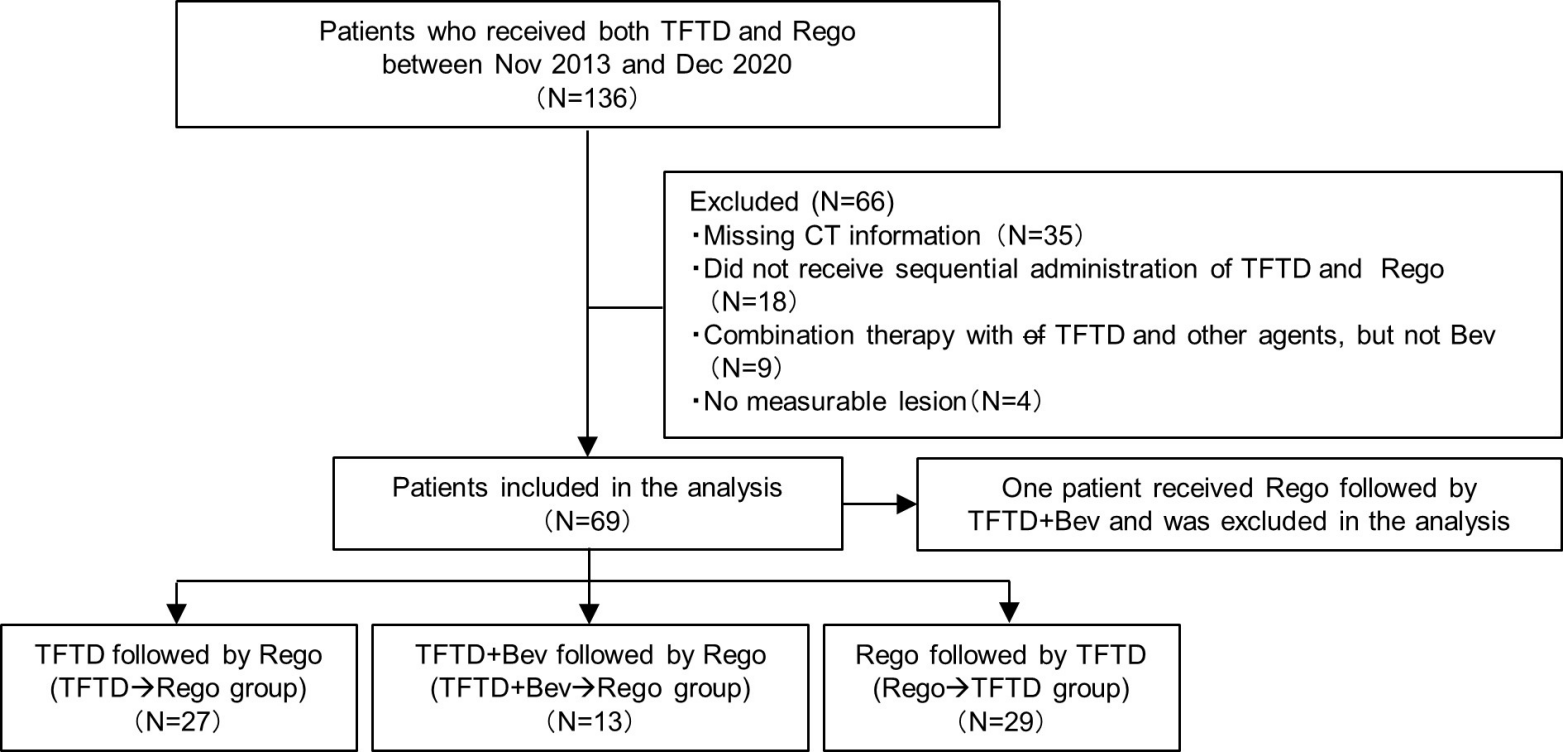
Flow diagram of patient selection.

Seventy patients satisfied the selection criteria (27 in TFTD→Rego group, 13 in TFTD+Bev→Rego group, 29 in Rego→TFTD group and 1 in Rego→TFTD+Bev group) (Fig 1). As there was only one patient in the Rego→TFTD+Bev group, this patient (group) was excluded from the analysis. There were no significant differences in the patient characteristics among the remaining three groups (Table 1); however, the numbers of patients with advanced disease, liver metastasis, and a short interval < 18 months from the initiation of first-line chemotherapy to the start of salvage chemotherapy were the highest in the TFTD+Bev→Rego group.

**Table 1:** Patient characteristics.

Characteristic	No. of patients (%)			p-value
	TFTD→Rego group	TFTD+Bev→Rego group	Rego→TFTD group	
	N = 27	N = 13	N = 29	
Age (years)				0.314
Median (range)	57 (33–81)	68 (27–86)	64 (37–79)	
Sex				0.434
Male	20 (74)	7 (54)	20 (69)	
Female	7 (26)	6 (46)	9 (31)	
ECOG PS				0.758
0	6 (22)	4 (31)	6 (21)	
1	21 (78)	9 (69)	22 (76)	
2	0 (0)	0 (0)	1 (3)	
Primary site				0.974
Right-sided^a^	8 (30)	4 (31)	8 (28)	
Left-sided^b^/rectum	19 (70)	9 (69)	21 (72)	
Histology				0.496
Adenocarcinoma				
Well- to moderately differentiated	25 (93)	12 (92)	25 (86)	
Poorly differentiated	1 (4)	0 (0)	3 (10)	
Mucinous	0 (0)	0 (0)	1 (3)	
Unknown differentiation	1 (4)	1 (8)	0 (0)	
*KRAS* status				0.824
Wild-type	13 (48)	7 (54)	14 (48)	
Mutant type	14 (52)	6 (46)	14 (48)	
Unknown	0 (0)	0 (0)	1 (3)	
*BRAF* status				0.835
Wild-type	14 (52)	7 (54)	14 (48)	
Mutant type	0 (0)	0 (0)	1 (3)	
Unknown	13 (48)	6 (46)	14 (48)	
Disease status				0.126
Advanced	17 (63)	11 (85)	15 (52)	
Recurrent	10 (37)	2 (15)	14 (48)	
Prior surgery for the primary tumor				0.427
Yes	21 (78)	9 (69)	25 (86)	
No	6 (22)	4 (31)	4 (14)	
Metastatic site				
Liver	18 (67)	12 (92)	23 (79)	0.181
Lung	18 (67)	9 (69)	16 (55)	0.573
Peritoneum	5 (19)	3 (23)	10 (35)	0.382
Lymph node	13 (48)	3 (23)	11 (38)	0.309
No. of metastatic sites				0.617
1–2	19 (70)	9 (69)	17 (59)	
≥3	8 (30)	4 (31)	12 (41)	
Prior chemotherapy regimens				0.863
2	14 (52)	6 (46)	16 (55)	
≥3	13 (48)	7 (54)	13 (45)	
Prior anti-VEGF antibody therapy				0.362
Yes	27 (100)	12 (92)	27 (93)	
No	0 (0)	1 (8)	2 (7)	
Prior anti-EGFR antibody therapy				0.692
Yes	11 (41)	4 (31)	13 (45)	
No	16 (59)	9 (69)	16 (55)	
Time from initiation of first-line chemotherapy				0.062
<18 months	6 (22)	9 (69)	9 (31)	
≥18 months	20 (74)	4 (31)	19 (66)	
Unknown	1 (4)	0 (0)	1 (3)	

Abbreviations: Right-sided^a^, Cecum, ascending colon, and proximal transverse colon

Left-sided^b^, distal transverse colon, descending colon, and sigmoid colon

Rego, regorafenib; TFTD, trifluridine/tipiracil; ECOG PS, Eastern cooperative oncology group Performance status; *KRAS*, v-Ki-ras2 Kirsten rat sarcoma viral oncogene homolog; *BRAF*, v-raf murine sarcoma viral oncogene homolog B1; VEGF, vascular endothelial growth factor; EGFR, epithelial growth factor receptor.

### Treatment exposure

Treatment modification (dose reduction, treatment interruption or, delay) was required in 11 patients (40.7%) and 26 patients (96.3%) during the precedent TFTD and subsequent regorafenib treatments in the TFTD→Rego group, in 9 patients (69.2%) and all 13 patients (100%) during the precedent TFTD+Bev and subsequent regorafenib treatments in the TFTD+Bev→Rego group, and in 24 (82.8%) and 15 (51.7%) patients during the precedent regorafenib and subsequent TFTD treatments in the Rego→TFTD group, respectively (S1 Table). The rate of initial dose reduction and the median relative dose intensity (RDI) in each treatment is shown in S1 Table.

The reason for treatment discontinuation during the precedent and subsequent treatments was disease progression in most patients of all three groups. Only one patient who received precedent regorafenib in the Rego→TFTD group discontinued due to hand-foot syndrome with regorafenib of Grade3 according to Common Terminology Criteria for Adverse Events version 4.0.

### Response

The ORRs and DCRs after each precedent and subsequent therapy in the three groups are shown in Table 2. PR was achieved in only one patient (3%) in subsequent TFTD in the Rego→TFTD group. As for the efficacy of each agent according to the treatment sequence, there were no significant differences in the DCR between the precedent TFTD in the TFTD→Rego group (11%) and subsequent TFTD in the Rego→TFTD group (21%) (p = 0.472), between the precedent regorafenib in the Rego→TFTD group (24%) and subsequent regorafenib in the TFTD→Rego group (22%) (p = 1.000), or between the precedent regorafenib in the Rego→TFTD group (24%) and subsequent regorafenib in the TFTD+Bev→Rego group (31%) (p = 0.713). There was no difference in the DCR achieved with subsequent regorafenib between the TFTD→Rego group (22%) and TFTD+Bev→Rego group (31%) (p = 0.700).

**Table 2:** Summary of the efficacy.

Group	TFTD→Rego group		TFTD+Bev→Rego group		Rego→TFTD group	
	(N = 27)		(N = 13)		(N = 29)	
Agent	Precedent TFTD	Subsequent Rego	Precedent TFTD+Bev	Subsequent Rego	Precedent Rego	Subsequent TFTD
ORR (number of pts, (%))	0 (0)	0 (0)	0 (0)	0 (0)	0 (0)	1 (3)
DCR (number of pts, (%))	3 (11)	6 (22)	3 (23)	4 (31)	7 (24)	6 (21)
median PFS (months)	1.87	1.93	2.07	1.47	1.87	1.73
	(1.60–2.07)	(1.53–2.33)	(1.63–2.67)	(1.00–2.17)	(1.37–2.40)	(1.00–2.80)
(95% CI)						
median T-PFS (months)	4.43		4.20		4.23	
	(3.83–5.60)		(3.47–5.37)		(2.90–5.53)	
(95% CI)						
median OS (months)	10.4		10.3		10.1	
	(4.83–12.8)		(4.77-not achieved)		(6.27–11.8)	
(95% CI)						
median TGR (%/month)	50.9	32.7	25.4	36.1	40.8	24.4
	(-6.1 to 205.7)	(-36.0 to 143.4)	(-4.2 to 132.9)	(8.9 to 49.0)	(-11.1 to 173.8)	(-55.6 to 302.9)
(range)						
Number of pts with decrease of TGR	20		6		19	
	(74%)		(46%)		(66%)	
median TGK (mm/month) (range)	8.76	7.79	7.49	9.92	8.02	7.20
	(-1.0 to 45.6)	(-2.8 to 83.3)	(-1.8 to 17.4)	(2.0 to 19.7)	(-6.2 to 22.1)	(-5.6 to 33.9)
Number of pts with decrease of TGK	15		4		17	
	(56%)		(31%)		(59%)	

Abbreviations: Rego, regorafenib; TFTD, trifluridine/tipiracil; Bev, bevacizumab; ORR, objective response rate; DCR, disease control rate; PFS, progression-free survival; T-PFS, two drugs PFS; OS, overall survival; TGR, tumor growth rate; TGK, tumor growth kinetics; pts, patients

### Survival

The PFS achieved with each therapy, the T-PFS and the OS in the three groups are summarized in Table 2. The median follow-up time of survivor was 7.7 months (range, 4.6–10.7 months) in the TFTD→Rego group, 10.0 months (range, 4.5–14.9 months) in the TFTD+Bev→Rego group and 5.8 months in the Rego→TFTD group. Events of progression disease on the image assessment were observed in all patients in each precedent and subsequent therapy in the three groups. Events of death were observed in 25 patients (92.6%) in the TFTD→Rego group, 7 patients (53.8%) in the TFTD+Bev→Rego group and 28 patients (96.6%) in the Rego→TFTD group.

Events of death were observed in 171 patients (77%) in the

regoraf eni b group and 247 patients (76%) in the TFTD group

**Fig 2:**
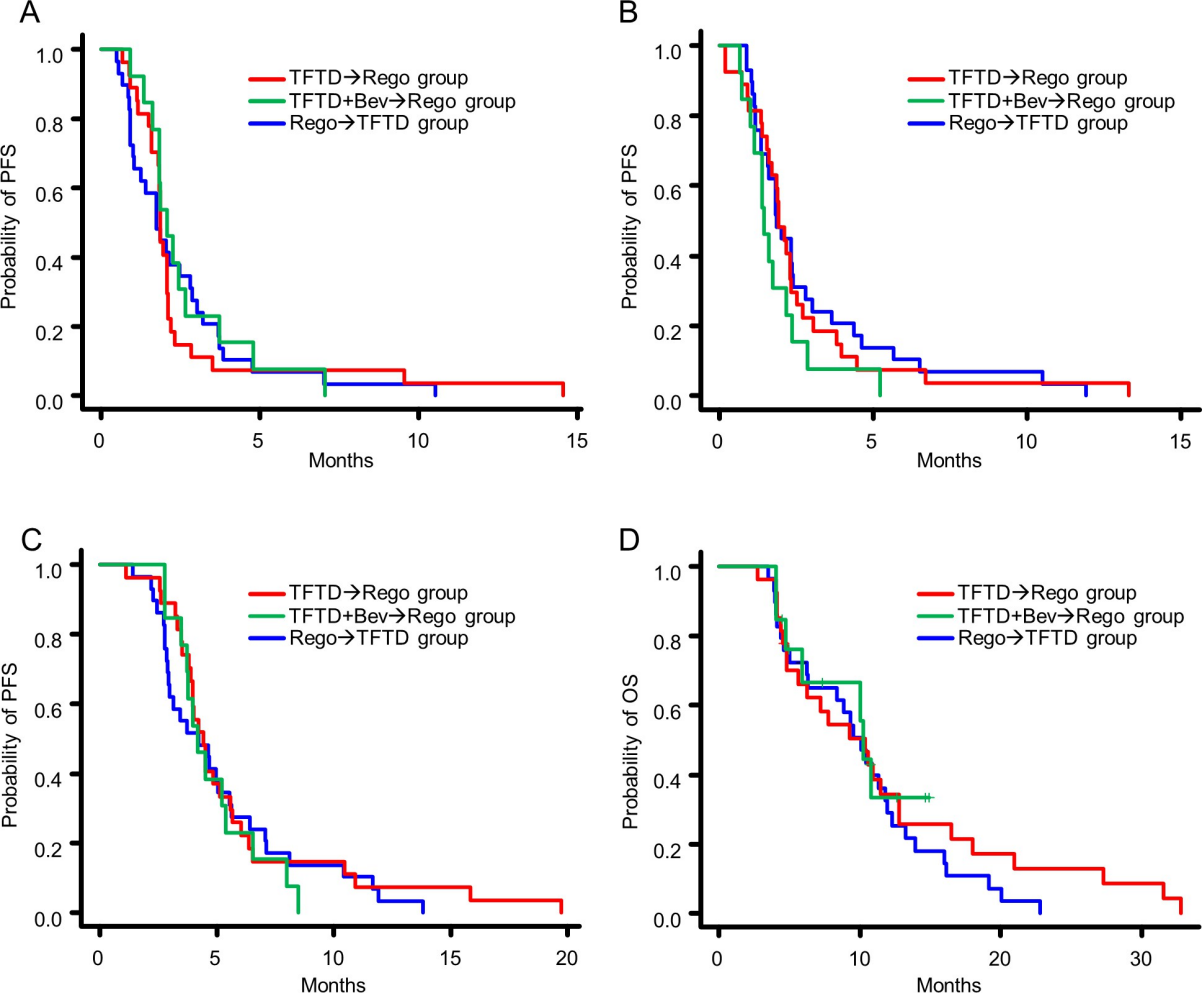
The efficacy of each agent and three groups. Kaplan-Meier plots of the (A) progression-free survival (PFS) after precedent and subsequent regorafenib therapy; (B) PFS after precedent and subsequent TFTD therapy; (C) PFS after sequential treatment with the two drugs (T-PFS); (D) overall survival (OS).

There was no difference in the PFS between the precedent TFTD in the TFTD→Rego group and subsequent TFTD in the Rego→TFTD group (median 1.87 vs. 1.73 months, HR, 1.04; 95% CI, 0.60–1.78; p = 0.899; Fig 2A), between the subsequent regorafenib in the TFTD-Rego group and precedent regorafenib in the Rego→TFTD group (median 1.93 vs. 1.87 months, HR, 1.02; 95% CI, 0.85–1.22; p = 0.822; Fig 2B), or between the subsequent regorafenib in the TFTD+Bev→Rego group and precedent regorafenib in the Rego→TFTD group (median 1.47 vs. 1.87 months, HR, 1.12; 95% CI, 0.98–1.28; p = 0.100; Fig 2B). There was no difference in the PFS between the subsequent regorafenib in the TFTD+Bev→Rego group and that in the TFTD→Rego group (median 1.47 vs. 1.93 months, HR, 1.51; 95% CI, 0.77–2.96; p = 0.226; Fig 2B). In addition, there were no significant differences in the T-PFS and OS among the three groups (Fig 2C and 2D, S2 Table).

### Tumor growth rate and tumor growth kinetics

**Fig 3:**
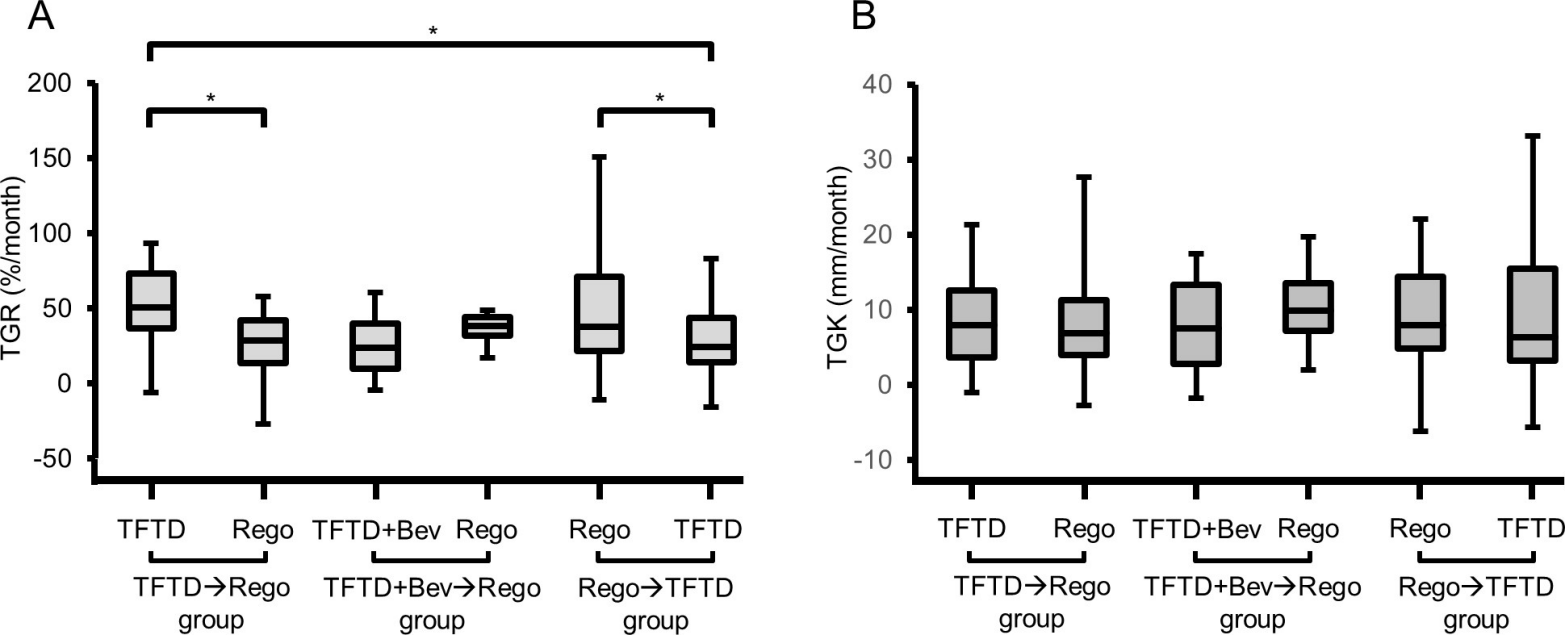
Distribution of the tumor growth rate and tumor growth kinetics. A. Distribution of the tumor growth rate. B. Distribution of the tumor growth kinetics. ^a^p-value<0.05.

The TGR and TGK during each therapy in the three groups are shown in Fig 3A and 3B, and summarized in Tables 2 and S3.

**Fig 4:**
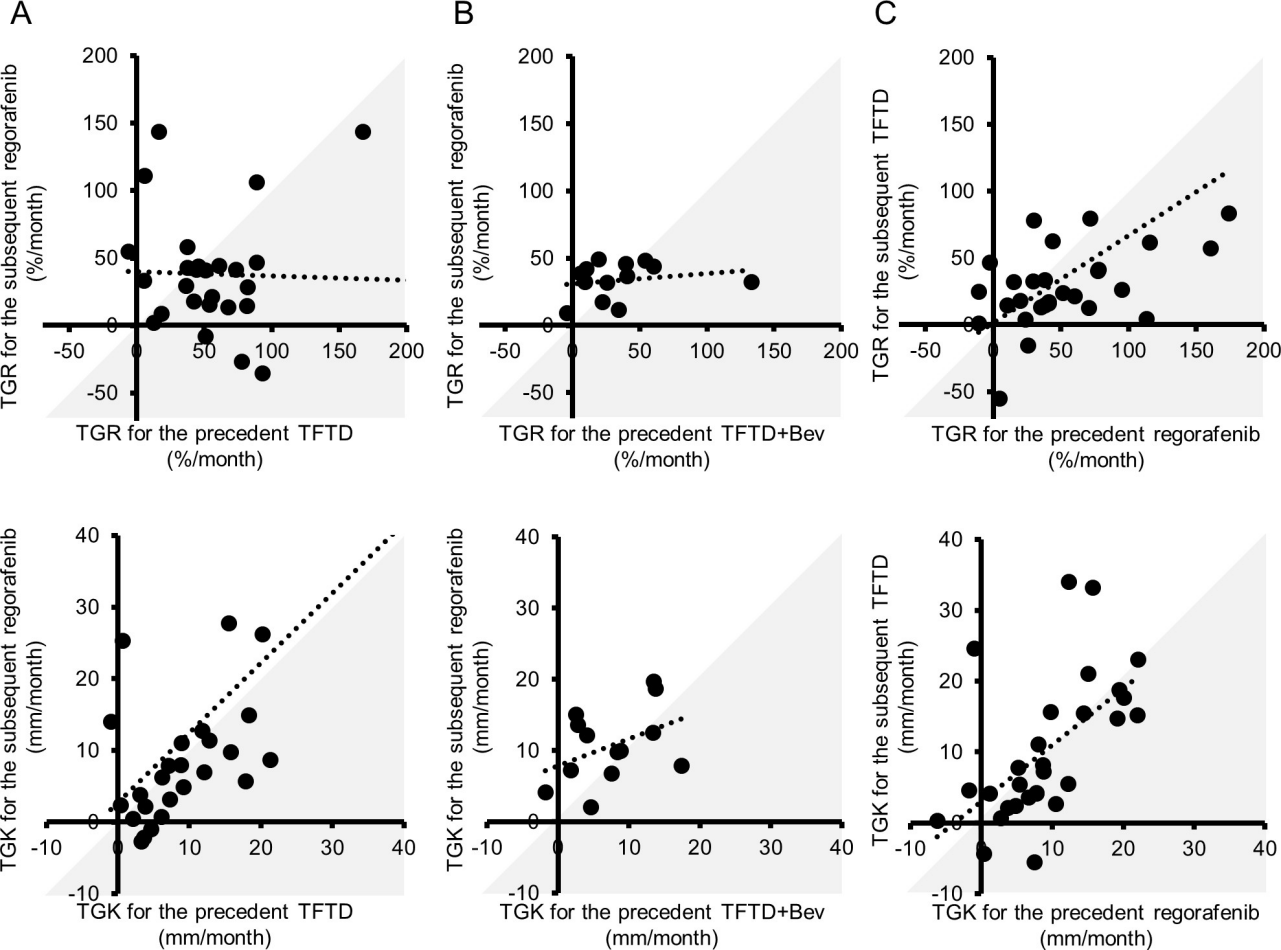
Distribution of the tumor growth rate and tumor growth kinetics in each pair of the precedent and subsequent agents. Distribution of the tumor growth rate (TGR) in the (A) TFTD→Rego group, (B) TFTD+Bev→Rego group, and (C) Rego→TFTD group; distribution of the tumor growth kinetics (TGK) in the (D) TFTD→Rego group, (E) TFTD+Bev→Rego group, and (F) Rego→TFTD group. The grey area indicates decrease in the TGR and TGF during the subsequent therapy as compared to the precedent therapy. Dot plot shows correlation between each pair of the TGR or TGK of the precedent agents and subsequent agents.

Comparing TGR between the precedent and subsequent therapies in each group, the TGR of the subsequent therapy decreased in 74% of patients (20/27) of the TFTD→Rego group (Fig 4A), 46% of patients (6/13) of the TFTD+BV→Rego group (Fig 4B), and 66% (19/29) of patients of the Rego→TFTD group (Fig 4C). The TGR during the subsequent regorafenib was significantly lower than that during the precedent TFTD in the TFTD→Rego group (p = 0.044), and TGR of the subsequent TFTD was significantly lower than the precedent regorafenib in the Rego→TFTD group (p = 0.027); conversely, there was no significant difference in the TGR achieved between the subsequent regorafenib and precedent TFTD+Bev in the TFTD+Bev→Rego group (p = 0.628). Comparison of the TGR achieved with each drug according to the treatment sequence revealed that the TGR during subsequent TFTD in the Rego→TFTD group was significantly lower than that during the precedent TFTD in the TFTD→Rego group (p = 0.014), while there was no statistically significant difference in the TGR between the subsequent TFTD in the Rego→TFTD group and precedent TFTD+Bev in the TFTD+Bev→Rego group (p = 1.000), indicating that the TGR during precedent TFTD+Bev was relatively smaller than that during the precedent TFTD in the TFTD→Rego group (p = 0.052). In contrast, there was no difference in the TGR between the subsequent regorafenib in the TFTD→Rego group and precedent regorafenib in the Rego→TFTD group (p = 0.227), or between the subsequent regorafenib in the TFTD+Bev→Rego group and that in the TFTD→Rego group (p = 0.798).

Comparison of the TGK among the three groups revealed that the TGK decreased in 56% (15/27) of patients in the TFTD→Rego group (Fig 4D), 31% of patients (4/13) in the TFTD+BV→Rego group (Fig 4E), and 59% of patients (17/29) in the Rego→TFTD group (Fig 4F). The TGK during the subsequent regorafenib was tended to be lower than that during the precedent TFTD in the TFTD→Rego group (p = 0.542), and TGK of the subsequent TFTD was also tended to be lower than that achieved with the precedent regorafenib in the Rego→TFTD group (p = 0.898); conversely, TGK during the subsequent regorafenib was tended to be higher than that during the precedent TFTD+Bev in the TFTD+Bev→Rego group (p = 0.068).

Comparison of the TGK achieved with each drug according to the treatment sequence revealed that TGK during subsequent TFTD in the Rego→TFTD group was tended to be lower than that during the precedent TFTD in the TFTD→Rego group (p = 0.871) although not significantly. TGK during precedent TFTD+Bev in the TFTD+Bev→Rego group was similar to that with the subsequent TFTD in the Rego→TFTD group (p = 0.536) and relatively smaller than that achieved with the precedent TFTD in the TFTD→Rego group (p = 0.458). In contrast, there was no difference in the TGK between the subsequent regorafenib in the TFTD→Rego group and precedent regorafenib in the Rego→TFTD group (p = 0.871), or between the subsequent regorafenib in the TFTD+Bev→Rego group and that in the TFTD→Rego group (p = 0.331).

## Discussion

To the best of our knowledge, this is the first study to assess the effect of TGR and TGK during crossover use of TFTD (with/without Bev) and regorafenib. RECIST is a validated and widely used criteria for evaluating the tumor responses to therapy, and the response is generally recognized and widely accepted as a short-term surrogate marker of the antitumor effect of the treatment. However, considering that TFTD and regorafenib yielded survival benefits despite quite low response rates, more detail evaluation of the response other than the RECIST, such as evaluation of the change in the tumor volume and/or size over a certain time, may be necessary for the short-term surrogate marker of the antitumor effect. TGR is a new assessment parameter that reflects the change in the tumor volume over time. Previous reports based on data from clinical trials have suggested that the TGR could detect the effect of treatment early [19–21] and that it was independently associated with the PFS [17, 19]. Furthermore, the TGR and TGK have also been used to assess tumor hyper-progression associated with immune checkpoint inhibitor therapy [22, 23]. We believe that the TGR and TGK could be used more widely in future clinical trials and research, although their precise clinical impacts still need to be investigated.

Both TFTD and regorafenib are approved as salvage treatments for refractory mCRC. In the RECOURSE trial, the median PFS was 2.0 months, the ORR was 1.6%, and the DCR was 44% in the TFTD arm [7]. In the CORRECT trial, the median PFS was 1.9 months, the ORR was 1.0%, and the DCR was 41% in the regorafenib arm [4]. Recently, combined TFTD plus Bev therapy has been used widely based on the results of a phase II study, which showed a median PFS of 4.6 months, ORR of 2.1%, and DCR of 67% [8]. The median PFS and ORR to the precedent therapy in each group in this study were consistent with the reports from these clinical studies, but the DCRs were lower than those reported in these clinical trials. The lower DCRs could be explained by the poor condition of the patients, as reflected by fewer patients with PS 0, and the lack of prespecified timing of the radiographic examinations in this retrospective study.

In regard to the efficacy of sequential treatment with TFTD and regorafenib administered in the forward or reverse sequence, it is generally considered that there are no differences in the conventional parameters of efficacy, whichever of the two agents is used first [9–12], while some reports showed that patients who received TFTD after regorafenib treatment showed a trend towards a longer PFS than those who received it prior to regorafenib [24]. Consistent with these reports, this study showed no significant difference in the OS, PFS, T-PFS, RR, or DCR between TFTD (with/without Bev) and regorafenib treatments administered either in the forward or the reverse sequence.

However, in detail, this study revealed that the TGR of the subsequent therapy was lower than that during the precedent therapy in both the TFTD→Rego and Rego→TFTD groups. The same tendency as with TGR was also observed with TGK. In contrast, both the TGR and TGK during precedent TFTD plus Bev were the lowest among the three precedent therapies of the three groups, suggesting that Bev might enhance the activity of TFTD. Moreover, the TGR and TGK during precedent TFTD plus Bev were similar to that during the subsequent TFTD in the Rego→TFTD group. Considering that Bev and regorafenib have similar anti-angiogenic effects, interaction between the precedent TFTD+BV and subsequent regorafenib might be the lowest in the TFTD+Bev→Rego group among the three groups. Additionally, previous reports showed that the crucial role of functional mitochondria on deactivation to antitumor effects of chemotherapeutic agents [25–27]. It suggests that the change of intracellular metabolism caused by mitochondria in cancer cells might affect the interaction of TFTD, regorafenib and Bev. Thus, this study suggests interaction between TFTD and regorafenib in terms of the TGR and TGK. However, because changes in the TGR and TGK along the natural course of tumor progression have not yet been clarified, it is not yet clear whether the observed effects on the TGR and TGK were caused by interaction between the precedent and subsequent therapies or by other reasons such as natural course. It is necessary to explore the interaction between regorafenib and TFTD to establish the best salvage chemotherapy strategy for mCRC in a prospective clinical trial.

This study has some limitations. First, because this study was retrospective and was limited to patients who received both TFTD and regorafenib sequentially, after excluding a substantial number of patients who received only one of these two drugs, there could have been a bias towards selection of patients who were in a relatively better condition who were preferentially treated with regorafenib and TFTD. Second, the intervals between imaging examinations were not set uniformly like in clinical trials; varying intervals between imaging examinations could have an influence on the estimated TGR and TGK. Third, the clinical impact of differences in the TGR and/or TGK on the survival remains unclear. Fourth, the shorter follow-up time in the TFTD+Bev→Rego group might affect the survival time that the follow up time in the TFTD+Bev→Rego group was shorter compared with other groups, because the efficacy of combination chemotherapy of TFTD plus Bev was reported in 2020 [8] but regorafenib was approved in 2013 and TFTD was approved in 2014 in Japan.

## Conclusion

This study showed no significant differences in the conventional parameters of efficacy between sequential treatment with TFTD (with/without Bev) and regorafenib administered in the forward or reverse sequence, although some interaction between the two drugs was suggested in the TGR and TGK in patients with mCRC refractory or intolerant to standard chemotherapy.

## Supporting information

S1 Table Summary of treatment exposure of each treatment in the three groups. (DOCX)

S2 Table The differences in the T-PFS and OS among the three groups. (DOCX)

S3 Table The differences in the TGR and TGK among the drugs in the three groups. (DOCX)
