# Supplementary material for: Influence of precedent drug on the subsequent therapy in the sequence of trifluridine/tipiracil with/out bevacizumab and regorafenib for unresectable or recurrent colorectal cancer
Source: PLoS One. 2022 Jun 2;17(6):e0269115. doi: 10.1371/journal.pone.0269115 (PMC9162345; doi:10.1371/journal.pone.0269115)
Supplement: S1 Table — (DOCX) [file pone.0269115.s001.docx]

S1 Table. Summary of treatment exposure of each treatment in the three groups.

| Group | TFTD→Rego group  (N=27) | | TFTD+Bev→Rego group  (N=13) | | Rego→TFTD group  (N=29) | |
| --- | --- | --- | --- | --- | --- | --- |
| Agent | Precedent TFTD | Subsequent  Rego | Precedent TFTD/Bev | Subsequent Rego | Precedent Rego | Subsequent TFTD |
| Number of pts with treatment modification (%) | 11 (40.7) | 26 (96.3) | 9 (69.2) | 13 (100) | 24 (82.8) | 15 (51.7) |
| Number of pts with initial dose reduction (%) | 1 (3.7) | 18 (66.7) | 2 (15.4) | 13 (100) | 12 (41.4) | 2 (6.9) |
| Median RDI (%)  (range) | 100  (64.4 to 100) | 53.3  (17.9 to 100) | 95.5/100  (63.9 to 100/64.5 to 100) | 50.0  (25.2 to 66.7) | 65.3  (22.5 to 100) | 98.8  (46.2 to 100) |

Abbreviations: Rego, regorafenib; TFTD, trifluridine/tipiracil; Bev, bevacizumab; RDI, relative dose intensity; pts, patients
