# Supplementary material for: Influence of precedent drug on the subsequent therapy in the sequence of trifluridine/tipiracil with/out bevacizumab and regorafenib for unresectable or recurrent colorectal cancer
Source: PLoS One. 2022 Jun 2;17(6):e0269115. doi: 10.1371/journal.pone.0269115 (PMC9162345; doi:10.1371/journal.pone.0269115)
Supplement: S2 Table — (DOCX) [file pone.0269115.s002.docx]

S2 Table. The differences in the T-PFS and OS among the three groups.

|  | TFTD→Rego group  vs  Rego→TFTD group | TFTD→Rego group  vs  TFTD+Bev→Rego group | TFTD+Bev→Rego group  vs  Rego→TFTD group |
| --- | --- | --- | --- |
| T-PFS  Median (months)  HR (95%CI)  p value | 4.43 vs 4.23  0.92 (0.71-1.21)  0.568 | 4.43 vs 4.20  1.25 (0.63-2.47)  0.521 | 4.20 vs 4.23  1.03 (0.87-1.22)  0.712 |
| OS  Median (months)  HR (95%CI)  p value | 10.4 vs 10.1  0.88 (0.66-1.16)  0.358 | 10.4 vs 10.3  1.01 (0.44-2.31)  0.985 | 10.3 vs 10.1  0.95 (0.76-1.17)  0.603 |

Abbreviations: Rego, regorafenib; TFTD, trifluridine/tipiracil; Bev, bevacizumab; T-PFS, two-drug progression free survival; OS, overall survival; HR, hazard ratio; CI, confidence interval
