# Supplementary material for: Influence of precedent drug on the subsequent therapy in the sequence of trifluridine/tipiracil with/out bevacizumab and regorafenib for unresectable or recurrent colorectal cancer
Source: PLoS One. 2022 Jun 2;17(6):e0269115. doi: 10.1371/journal.pone.0269115 (PMC9162345; doi:10.1371/journal.pone.0269115)
Supplement: S3 Table — (DOCX) [file pone.0269115.s003.docx]

S3 Table. The differences in the TGR and TGK among the drugs in the three groups.

|  | Comparison factor | p-value |
| --- | --- | --- |
| TGR | Comparison of precedent and subsequent drug in each group |  |
|  | TFTD vs Regorafenib in TFTD→Rego group | 0.044 |
|  | Regorafenib vs TFTD in Rego→TFTD group | 0.027 |
|  | TFTD+Bev vs Regorafenib in TFTD+Bev→Rego group | 0.628 |
|  | Comparison of each drug according to the treatment sequence |  |
|  | Precedent TFTD in TFTD→Rego group vs  Subsequent TFTD in Rego→TFTD group | 0.014 |
|  | Subsequent TFTD in Rego→TFTD group vs Precedent TFTD+Bev in TFTD+Bev→Rego group | 1.000 |
|  | Precedent TFTD in TFTD→Rego group vs  Precedent TFTD+Bev in TFTD+Bev→Rego group | 0.052 |
|  | Subsequent regorafenib in TFTD→Rego group vs  Precedent regorafenib in Rego→TFTD group | 0.227 |
|  | Subsequent regorafenib in TFTD→Rego group vs  Subsequent regorafenib in TFTD+Bev→Rego group | 0.798 |
| TGK | Comparison of precedent and subsequent drug in each group |  |
|  | TFTD vs Regorafenib in TFTD→Rego group | 0.542 |
|  | Regorafenib vs TFTD in Rego→TFTD group | 0.898 |
|  | TFTD+Bev vs Regorafenib in TFTD+Bev→Rego group | 0.068 |
|  | Comparison of each drug according to the treatment sequence |  |
|  | Precedent TFTD in TFTD→Rego group vs  Subsequent TFTD in Rego→TFTD group | 0.871 |
|  | Subsequent TFTD in Rego→TFTD group vs Precedent TFTD+Bev in TFTD+Bev→Rego group | 0.536 |
|  | Precedent TFTD in TFTD→Rego group vs  Precedent TFTD+Bev in TFTD+Bev→Rego group | 0.458 |
|  | Subsequent regorafenib in TFTD→Rego group vs  Precedent regorafenib in Rego→TFTD group | 0.871 |
|  | Subsequent regorafenib in TFTD→Rego group vs  Subsequent regorafenib in TFTD+Bev→Rego group | 0.331 |

Abbreviations: Rego, regorafenib; TFTD, trifluridine/tipiracil; Bev, bevacizumab; TGR, Tumor growth rate; TGK, tumor growth kinetics
